# The Effect of Different Foam Concentrations on Sperm Motility in Japanese Quail

**DOI:** 10.4061/2010/564921

**Published:** 2010-11-08

**Authors:** Avishek Biswas, O. S. Ranganatha, Jag Mohan

**Affiliations:** ^1^Division of Physiology and Reproduction, Central Avian Research Institute, Izatnagar, Bareilly 243122, UP, India; ^2^Defence Institute of High Altitude Research, DRDO, C/o 56 APO, Leh 194101, J&K, India

## Abstract

A study was conducted to determine the effect of foam extract on sperm motility in the male Japanese quail (*Coturnix coturnix japonica*). Adult male quails (<12 weeks) of heavy body weight strain were housed in individual cages and divided into 5 groups according to the size of their cloacal glands. The data indicated that the size of the cloacal gland was positively correlated with the frequency of foam secretion and total foam production. One gram of freshly collected clean foam was mixed with 1.0 mL of normal saline and homogenized for 10 minutes. After centrifugation at 35 000 rpm, the supernatant was used as 100% foam extract. The extract was diluted to 1:40, 1:20, 1:10, and 1:4 with normal saline to produce 2.5, 5.0, 10, and 25% foam extracts, respectively. 5% foam extract enhanced sperm survival at room temperature (30°–35°C) for 2 to 3 hrs, whereas higher concentrations (10% and above) suppressed sperm motility. From this study, it may be concluded that foam secretion and quantity of foam are directly proportional to the size of the cloacal gland and that the foam enhances and prolongs sperm motility, in vitro at an optimum concentration of 5%.

## 1. Introduction

In domestic fowlsecondary sexual characteristics (comb and wattle) have been used as external indicators of sex and stage of sexual maturity. Growth of combs and wattles is induced by androgens [1], which in male Japanese quail also induce development of the cloacal gland [2–4], an organ that is specific to members of the *Coturnix *genus. Under the influence of testosterone, the cloacal gland of sexually active male Japanese quail constantly produces meringue-like foamy materials irrespective of time of the day [2]. There is a positive correlation between the size of the cloacal gland, the weight of the testes, sexual activity, and fertility [5–8]. 

The role of the foamy material in Japanese quail reproduction has been the subject of conflicting reports during the last three and half decades [9–11]. Some workers reported that quail foam might have a beneficial effect on the fertilizing ability of spermatozoa [10–12], whereas others reported that foam might be detrimental to quail spermatozoa [11].

Most of the research on foam in male Japanese quail has been related to quantity, time of secretion, and bacterial count [13]. No information is available on the effects of different concentrations of cloacal foam extract on sperm motility in male Japanese quail or of the possible role of foam in other aspects of reproduction in Japanese quail. The research described here was carried out to investigate the effects of cloacal foam extract on sperm motility in male Japanese quail.

## 2. Materials and Methods

### 2.1. Housing and Rearing of Birds

One hundred and fifty (150) male Japanese quails of heavy body weight strain more than 12 weeks of age and from the same hatch were procured from the experimental quail farm CARI, Izatnagar, India. The birds were maintained in individual cages under uniform husbandry conditions (14 hrs light/day, 20–30°C) and provided with breeder ration and water *ad libitum*. For the purpose of analysis, birds were arbitrarily divided into 5 groups (30 birds in each) according to the cloacal gland size. The same technical personnel provided food and water and collected data from the birds during the course of the experiment. All the experiments were conducted strictly in accordance with the guidelines of ‘Institutional Animal Ethics Committee' (IAEC).

### 2.2. Collection of Foam

The size of the cloacal glands was measured as described by Siopes and Wilson [14] and was recorded as an index. Frequency of foam release was determined by observing the number of foamy masses in the faecal trays under each bird at 6 AM, 12 noon, and 6 PM. Foam produced by each group of birds was pooled, and its weight was determined immediately using an electronic analytical balance [8]. Foam for preparation of foam extract was collected by gently squeezing the cloacal gland on either side with fingers and thumb [15] and was stored in airtight glass bottles to prevent evaporation.

### 2.3. Preparation of Extract

One gram of fresh, clean foam free from faecal contamination was mixed with 1.0 ml of normal saline (0.89% w/v NaCl) and homogenized for 10 minutes. Subsequently, the mixture was centrifuged at 35000 rpm in “ultracentrifuge” (himac CP80*β*) for 30 minutes. The sediment was discarded and the supernatant was used as 100% foam extract. It was diluted to 1:40, 1:20, 1:10, and 1:4 with normal saline to produce 2.5, 5.0, 10, and 25% foam extract, respectively.

### 2.4. Collection of Semen

Semen was collected as per the procedure described by Lake [16]. To prevent contamination with foam and watery fluid, special care was taken. Just before the collection of semen, foam was removed from the cloacal gland. The cloacal area was then cleaned gently with tissue paper. Each adult mail quail was gently picked up with one person holding the legs apart and another collecting the semen discharge after gently massaging the lumber region of the bird three to four times with the hand. From the pooled semen, 10 *μ*l semen was diluted with 50 *μ*l normal saline (0.89%, w/v; NaCl) and kept as control. Then, each 10 *μ*l semen was diluted with 50 *μ*l of different concentrations of foam extracts and mixed well. The diluted semen was aspirated and stored in small glass tubes for further observation. 

Immediately after dilution, the percentage of motile spermatozoa was determined by compound microscope at 10X magnification after placing a coverslip over 2–3 mm drop of diluted semen on a microscope slide. The motility was recorded at 30 minute intervals up to 3.0 hours of storage (at room temperature; 18°C) to study the effect of foam on survival of spermatozoa.

### 2.5. Statistical Analysis

The data were analyzed by analysis of variance [17], and means were compared by Duncan multiple range test [18].

## 3. Results

The mean cloacal gland index, frequency of foam secretion (in 24 hrs), foam weight (mg), and the effect of different concentrations of fresh foam extract on the motility of quail vas deferens spermatozoa are presented in Tables 1 and 2.

The mean gland size index (mm^2^) increased in linear fashion (*P* < .01) from 232.26 ± 1.24 in Group 1 to 480.82 ± 5.06 in Group 5. The frequency of foam secretion was significantly (*P* < .01) higher in Group 5 (22.59 ± 1.36) than in the remaining groups. No significant difference was observed between Groups 1 and 2 and among Groups 2, 3, and 4. However, the frequency of foam secretion was significantly (*P* < .01) higher in Groups 3 (17.73 ± 0.93) and 4 (17.61 ± 1.09) than in Group 1 (14.75 ± 0.71).

The quantity of foam collected in Groups 1 to 5 was 13.30 ± 1.05, 18.20 ± 0.88, 22.45 ± 1.67, 23.26 ± 1.19, and 25.40 ± 1.38, respectively. The means of Groups 3 to 5 were significantly (*P* < .01) higher than those of Groups 1 and 2. The smallest quantity of foam was from Group 1 and was significantly (*P* < .01) lower than that from Group 2.

Immediately after dilution (0 hr), the sperm motility of the semen diluted with foam extract was significantly (*P* < .01) higher than that of semen diluted with normal saline (control). Among the foam-diluted semen samples, the percentage of motility in the 25% foam extract was significantly (*P* < .01) less than in the other dilutions. After 30 minutes storage of sample at room temperature, the motility in 2.5, 5.0, and 10% foam extracts was significantly (*P* < .01) higher than in the control and 25% foam extract groups. At 1.0 hr storage, maximum motility (80.00 ± 2.88) was observed in the 5% foam extract group followed by 2.5%  (70.00 ± 2.58) and 10%  (69.16 ± 3.27). These three groups showed significantly (*P* < .01) higher motility than in the control (54.16 ± 4.54). Minimum motility was observed in the 25% foam extract group. After 1.5 hrs of storage, the motility in the 5.0% foam extract was 71.66 ± 4.01  (%), and it was significantly (*P* < .01) higher than all other groups. A similar pattern of motility was found after 2.0 hr of storage. Throughout the experiment semen diluted with 5.0% foam extract had the highest percentage of motile spermatozoa (Table 2).

## 4. Discussion

In sexually active male Japanese quail, foam is synthesized in the cloacal gland. In this study, birds were categorized into groups based on the size of their cloacal glands. The mean frequency of foam secretion over 24 hrs increased from Group 1 (smallest cloacal glands) to Group 5 (largest cloacal glands). This difference was highly significant (*P* < .01). These results indicated that the frequency of foam secretion is positively correlated with size of the cloacal glands. This observation is in agreement with the report of Mohan et al. [8]. However, Ikeda and Taji [19] reported that the rate of foam secretion is random. 

The quantity of foam produced (mg/bird) followed the same trend as that of frequency of foam secretion. The increase in the quantity of foam production is consistent with the increase in gland size suggesting that the amount of foam produced is closely associated with size of the cloacal gland. Moudgal and Mohan [20] also reported a similar relationship between foam production and size of cloacal glands.

In this study, the motility of spermatozoa was studied in quail semen collected from the bird and diluted with normal saline (control) or several concentrations of cloacal gland foam extract. At collection, the majority of the spermatozoa were agglutinated. When the semen was diluted with normal saline (control), dispersion of the agglutinated sperm was very slow and incomplete. The agglutinated spermatozoa were easily dispersed when the semen was diluted with foam extract. This effect of foam extract was observed at all concentrations, 2.5, 5.0, 10, and 25%. Maximum motility was observed in semen diluted with 5.0% foam extract, and at this dilution motility was significantly (*P* < .01) higher than control and other treatments at all time intervals. Even after 3 hrs of storage of sample at room temperature, the motility of spermatozoa was better in the 5.0% foam extract than in all other dilutions. These results show that quail foam has a favourable effect on the survival of spermatozoa *in vitro*. Studies on quails concerning the relationship between different foam concentrations and semen motility have not been published so far, and to our knowledge, the present report is the first one on this aspect.

To rule out the possibility that variation in osmolarity may be responsible for the differences in motility between treatments, the osmolarity of all the diluents was determined. The osmolarities of normal saline, 2.5, 5.0, 10, and 25% foam extracts were 291, 298, 300, 306, and 309 milliosmole, respectively. It is unlikely that these minor differences in osmolarity would have a major effect on sperm motility. Furthermore, slight differences were observed in the pH of different diluents. It is suggested that the favorable effect of quail foam is due to the presence of some active ingredient present in the water-soluble fraction of foam. It is interesting that at higher concentration (25%) the foam extract had an inhibiting effect on sperm motility.

## 5. Conclusion

From this study, it may be concluded that the frequency of foam secretion and quantity of foam production are directly proportional to the size of the cloacal gland and that the effect of quail foam on sperm motility, *in vitro,* is dose dependent. At optimum concentration (5.0%), the foam extract enhances and prolongs sperm motility, whereas at higher concentration (10% and above), the foam extract suppresses sperm motility.

## Figures and Tables

**Table 1:** Cloacal gland index, frequency of foam secretion, and quantity of foam in Japanese quail (mean ± S.E.M., *n* = 12).

Group	Cloacal gland size index	Frequency of foam deposition				Quantity of foam
	(mm^2^)	6 AM	12 noon	6 PM	Total	(mg/bird)
1	232.26 ± 1.24^a^	5.86 ± 0.27	3.83 ± 0.24	5.06 ± 0.43	14.75 ± 0.71^a^	13.30 ± 1.05^a^
2	324.27 ± 0.98^b^	6.66 ± 0.35	4.10 ± 0.27	5.10 ± 0.24	15.86 ± 0.55^ab^	18.20 ± 0.88^a^
3	377.50 ± 0.84^c^	7.54 ± 0.34	4.84 ± 0.31	5.35 ± 0.50	17.73 ± 0.93^b^	22.45 ± 1.67^b^
4	421.64 ± 2.21^d^	7.60 ± 0.43	5.04 ± 0.40	4.96 ± 0.37	17.61 ± 1.09^b^	23.26 ± 1.19^c^
5	480.82 ± 506^e^	8.90 ± 0.34	5.56 ± 0.34	8.08 ± 1.01	22.59 ± 1.36^c^	25.40 ± 1.38^d^

Values bearing different superscripts within the columns differ significantly (*P* < .01).

**Table 2:** Effect of foam extract on sperm motility (percentage of motile sperm) at different time intervals at room temperature (mean ± S.E.M., *n* = 30).

Duration							
	0 hr	0.5 hr	1.0 hr	1.5 hr	2.0 hr	2.5 hr	3.0 hr
NS*	36.66 ± 2.10^a^	51.66 ± 4.77^a^	54.16 ± 4.54^b^	42.50 ± 4.95^b^	22.50 ± 4.95^b^	5.00 ± 1.82^b^	00.00 ± 0.00^a^
2.5% FE**	90.00 ± 0.00^c^	81.66 ± 2.78^b^	70.00 ± 2.58^c^	43.33 ± 6.00^b^	22.50 ± 3.59^b^	7.50 ± 2.14^b^	00.00 ± 0.00^a^
5% FE	90.00 ± 0.00^c^	87.50 ± 1.70^b^	80.00 ± 2.88^d^	71.66 ± 4.01^c^	52.50 ± 5.88^c^	41.66 ± 7.81^d^	15.00 ± 2.58^c^
10% FE	85.83 ± 1.53^c^	77.50 ± 2.14^b^	69.15 ± 3.27^c^	47.50 ± 7.27^b^	30.83 ± 2.38^b^	22.50 ± 2.14^c^	10.83 ± 1.53^b^
25% FE	63.33 ± 4.01^b^	49.16 ± 4.36^a^	24.16 ± 3.00^a^	15.00 ± 2.58^a^	09.16 ± 1.53^a^	00.00 ± 0.00^a^	00.00 ± 0.00^a^

Values bearing different superscripts within the columns differ significantly (*P* < .01).

NS*: Normal saline, FE**: Foam extract.
